# Temperature-Defined Heat-Alert-Threshold Days and Acute Myocardial Infarction Admissions and Mortality: A Nationwide Hungarian Registry-Based Cohort Study

**DOI:** 10.3390/ijerph23081010

**Published:** 2026-08-02

**Authors:** Csaba Bálint, Ali Abbas Rahi Al-Murshedi, Ammar Mahmood Jaber, Annamária Pakai, Zsófia Verzár

**Affiliations:** 1 Doctoral School of Health Sciences, Faculty of Health Sciences, University of Pécs, 7621 Pécs, Hungary; 2 Faculty of Health Sciences, Institute of Emergency Care, Pedagogy of Health and Nursing Sciences, University of Pécs, 7621 Pécs, Hungary; 3 Medical School, Heart Center, University of Pécs, 7624 Pécs, Hungary

**Keywords:** heat-alert-threshold day, heat wave, heat-health action plan, acute myocardial infarction, STEMI, NSTEMI, cumulative mortality, cardiovascular epidemiology, Hungary

## Abstract

**Highlights:**

**Public health relevance—How does this work relate to a public health issue?**

- Heat waves and extreme heat events, increasingly frequent due to climate change, are established triggers for acute cardiovascular events, including myocardial infarction (AMI).
- Official heat alerts issued by public health authorities represent real-world policy interventions aimed at mitigating population-level risks during periods of elevated temperature extremes. In Hungary, temperature-defined heat-alert-threshold days represent the operational criterion used to trigger the national heat-health action plan.

**Public health significance—Why is this work of significance to public health?**

- This nationwide Hungarian study found that heat-alert-threshold days were associated with fewer recorded AMI admissions during summer (aIRR 0.93). This unexpected finding should not be interpreted as evidence of a protective effect of heat exposure and may reflect multiple factors, including under-ascertainment of out-of-hospital events and residual confounding.
- Heat-alert-threshold exposure was not associated with long-term post-AMI mortality among hospitalized patients (adjusted hazard ratio 0.96). This finding should not be interpreted as evidence that heat exposure has no adverse cardiovascular consequences at the population level.

**Public health implications—What are the key implications or messages for practitioners, policy makers and/or researchers in public health?**

- The present findings support continued surveillance of cardiovascular events during periods meeting the national heat-alert temperature threshold while emphasizing the need for more comprehensive exposure assessment.
- Future studies should integrate administrative heat-alert records, meteorological data, emergency medical services, out-of-hospital deaths, and hospital registries to better evaluate the public-health impact of heat-alert systems.

**Abstract:**

Background: Although extreme heat is associated with adverse cardiovascular outcomes, the relationship between heat-alert-threshold days, acute myocardial infarction (AMI) admissions, and post-AMI mortality remains uncertain. We aimed to evaluate the association between temperature-defined heat-alert-threshold days and (i) daily AMI admissions and (ii) cumulative all-cause mortality after hospitalized AMI in Hungary. Methods: We conducted a nationwide registry-based study using data from the Hungarian Myocardial Infarction Registry (HMR). All AMI admissions between 1 January 2018 and 31 December 2019 were eligible, with mortality follow-up through 16 June 2021. Heat-alert-threshold days were defined using the temperature criterion applied in the Hungarian national heat-health action plan (daily mean temperature ≥25 °C). AMI admissions were analyzed using adjusted quasi-Poisson regression models and cumulative mortality using stratified Cox proportional hazards models. This operational temperature threshold was used as the exposure definition and does not represent linkage to administrative heat-alert declarations. Results: The cohort included 30,883 AMI events from 29,596 unique patients (mean age, 67.2 [SD 12.8] years; 60.3% male). Patients admitted on heat-alert-threshold days had baseline clinical characteristics similar to those admitted on non-alert days (all standardized mean differences <0.10). Heat-alert-threshold days were associated with fewer recorded AMI admissions during summer (adjusted incidence rate ratio [aIRR] 0.93, 95% CI 0.90–0.97). In contrast, no association was observed between heat-alert-threshold exposure and subsequent all-cause mortality after AMI hospitalization (adjusted hazard ratio [aHR] 0.96, 95% CI 0.87–1.05). These findings remained consistent across sensitivity analyses. Conclusions: In this nationwide Hungarian registry-based study, temperature-defined heat-alert-threshold days were associated with fewer recorded AMI admissions during summer but not with differences in post-AMI mortality among hospitalized patients. The observed inverse association should not be interpreted as evidence of a protective effect of heat exposure. Alternative explanations include behavioral adaptation, delayed care-seeking, exposure misclassification, residual confounding, and the possibility of unmeasured out-of-hospital cardiovascular events, which were not captured by the registry. Because administrative heat-alert declarations were not linked to the analytic dataset, the findings should be interpreted as associations with temperature-defined heat-alert-threshold days rather than evaluations of the effectiveness of the Hungarian heat-alert system.

## 1. Introduction

Cardiovascular diseases remain the leading cause of mortality worldwide, with acute myocardial infarction (AMI) representing a major contributor to morbidity, hospital admissions, and long-term mortality [1,2,3,4,5]. Concurrently, anthropogenic climate change has increased the frequency, duration, and intensity of heat waves since the 1950s, raising concerns regarding their cardiovascular health effects [6,7,8,9].

Heat waves induce several physiological responses that may increase the risk of acute myocardial infarction (AMI), including dehydration-related hemoconcentration, increased myocardial oxygen demand, autonomic imbalance, systemic inflammation, and prothrombotic changes in hemostasis [8,10,11,12]. Epidemiological studies have linked high ambient temperature to increased cardiovascular morbidity and mortality, although the magnitude, timing, and direction of these associations vary across populations, exposure definitions, lag structures, and analytical approaches [11,13,14,15,16,17,18]. Some studies have reported stronger effects among older adults and during more intense heat events [11,13,14,15]. However, evidence regarding cumulative post-AMI mortality remains limited and heterogeneous [11,16,17,18].

From a public-health perspective, meteorological conditions reaching official heat-alert thresholds may represent a particularly relevant exposure because they trigger public health messaging, healthcare-system preparedness, and different response mechanisms. However, the present study evaluates the temperature criterion underlying the Hungarian heat-health action plan rather than the implementation or effectiveness of administrative heat-alert declarations. Across Europe, heat-health action plans commonly use predefined meteorological thresholds to issue tiered alerts intended to reduce heat-related health risks. In Hungary, the National Public Health Center (NNK) uses a daily mean ambient temperature of 25 °C as the criterion for issuing first-tier (yellow) heat alerts. This threshold should be interpreted as a country-specific operational definition used within the Hungarian heat-health action plan rather than as a universal physiological cutoff for extreme heat [19].

To our knowledge, few nationwide registry-based studies have evaluated temperature-defined heat-alert-threshold exposure in relation to both AMI admissions and cumulative post-AMI mortality. In the present study, exposure was operationalized using the predefined temperature criterion employed by the Hungarian National Public Health Center (NNK) to define heat-alert-threshold days.

Previous studies have often relied on modeled temperatures, single-city datasets, or population-level mortality analyses, with limited linkage to nationwide cardiovascular registries. Previous studies have generally reported increased cardiovascular morbidity during periods of extreme heat; however, results have varied according to exposure definition, study design, climatic region, and outcome selection [11]. We therefore used data from the Hungarian Myocardial Infarction Registry to examine whether heat-alert-threshold days, operationalized using the NNK temperature criterion, were associated with daily AMI admission rates and cumulative all-cause mortality after AMI hospitalization. We additionally explored whether these associations differed according to demographic and clinical characteristics.

## 2. Materials and Methods

### 2.1. Study Design and Reporting

We conducted a nationwide retrospective study combining a county-day time-series analysis of AMI admissions with a patient-level cohort analysis of cumulative all-cause mortality after the index AMI event. The study population comprised all AMI events recorded in the Hungarian Myocardial Infarction Registry (HMR) [20] between 1 January 2018 and 31 December 2019, with mortality follow-up extending through 16 June 2021. The study followed the RECORD reporting guideline [21], an extension of the STROBE statement for observational studies [22]. The completed RECORD checklist is provided as Supplementary Materials.

### 2.2. Data Sources

The Hungarian Myocardial Infarction Registry (HMR) is a nationwide prospective database that captures hospitalized AMI events in Hungary. Established in 2010 and maintained by the National Institute of Cardiology, the registry collects standardized electronic case-report data on patient demographics, clinical presentation, comorbidities, in-hospital management, and outcomes. Longitudinal follow-up is enabled through deterministic linkage to the National Health Insurance Fund database using unique patient identifiers, allowing follow-up for up to 10 years after the index event. AMI events are classified according to the registry’s standard diagnostic criteria, and the HMR has been described in detail previously [23]. The analytic dataset used in this study is publicly available in the Mendeley Data repository (DOI: 10.17632/2v7n2r3xch.1) [20]. The study used fully anonymized retrospective registry data and was approved by the University of Pécs ethical committee (8085/PTE2020), which confirmed that additional informed consent was not required under Hungarian regulations.

### 2.3. Study Population

All AMI events recorded in the HMR between 1 January 2018 and 31 December 2019 were eligible for inclusion. Eligible events met the registry’s standard inclusion criteria: age ≥ 18 years, documented AMI (ICD-10 codes I21–I22), and complete demographic data. For patient-level survival analyses, the index event was defined as the first observed AMI per patient during the study period. Of 30,883 AMI events, 29,596 represented unique-patient index events. Minor inconsistencies identified during data cleaning were resolved prior to analysis without excluding any events.

### 2.4. Geographical Linkage and Exposure Definition

Each patient’s residential county (one of 19 counties plus the capital city of Budapest) was linked to the nearest OMSZ (Hungarian Meteorological Service) meteorological station among the ten stations covering the country (Budapest, Debrecen, Keszthely, Miskolc, Nyíregyháza, Pécs, Sopron, Szeged, Szombathely, and Túrkeve). Each county was mapped consistently to only one station; stations served between one and four counties (median: two). A heat-alert-threshold day was defined a priori as a calendar day on which the daily mean ambient temperature at the assigned station was ≥25 °C, the criterion used by the NNK to issue first-tier (yellow) heat alerts under the national heat-health action plan [19,24]. This is a country-specific, policy-defined operational threshold rather than a universal physiological cutoff for extreme heat, and we use the term “heat-alert-threshold day” throughout to make this distinction explicit.

Because actual administrative alert declarations were not linked directly to the analytic dataset, the exposure should be understood as an operational, temperature-defined indicator rather than a measure of administrative alert issuance. The binary heat-alert-threshold indicator served as the primary exposure variable. As a sensitivity definition, we additionally constructed a sustained-heat indicator, set to one if both the same-day mean temperature and the three-day rolling mean (current and previous two days) exceeded 25 °C, to capture extended heat episodes rather than isolated hot days. Because every patient on a given day in each county shared the same exposure status by construction, exposure was effectively assigned at the station-day rather than the individual-patient level. The primary admission analysis was therefore conducted at the county-day rather than the patient level, with statistical inference based on cluster-robust standard errors at the meteorological-station level and on a station-level cluster bootstrap to provide inference robust to the small number of clusters (*n* = 10) [25].

### 2.5. Outcomes

The two co-primary outcomes were (i) daily count of recorded AMI admissions per county and (ii) all-cause mortality after the index AMI hospitalization. Mortality follow-up was measured from index admission to death from any cause or administrative censoring on 16 June 2021, corresponding to the most recent vital-status update available at the time of analysis. Mortality dates were obtained through deterministic linkage to the Central Death Registry. Twenty-eight in-hospital deaths recorded on the same calendar day as admission (follow-up time of zero days) were retained. For time-stratified analyses requiring strictly positive follow-up, all follow-up times were uniformly increased by 0.5 days, consistent with standard survival-analysis practice. The admission outcome reflects recorded AMI hospitalizations and does not capture out-of-hospital cardiac fatalities or silent infarctions. Similarly, the mortality analysis evaluated post-AMI mortality according to exposure status on the admission day and should not be interpreted as a direct estimate of population-level heat-related cardiovascular mortality.

### 2.6. Statistical Analysis

#### 2.6.1. Cohort Description

Baseline characteristics were summarized as means with standard deviations for continuous variables and counts with percentages for categorical variables, stratified by exposure status on the index admission day. Because *p*-values may be misleading in large observational datasets, standardized mean differences (SMDs) were used as the primary measure of covariate imbalance. An absolute SMD < 0.10 was considered indicative of meaningful imbalance, consistent with current cardiovascular epidemiology practice [26].

#### 2.6.2. AMI Admissions Analysis

AMI admissions were analyzed at the county-day level. A panel of 14,600 county-days (20 administrative units × 730 days) was constructed, with each county-day containing the number of AMI admissions and the corresponding meteorological exposure. The primary analysis used quasi-Poisson regression with admission count as the outcome and the binary heat-alert-threshold indicator as the exposure, restricted to the summer months (June–August). Models were adjusted for day-of-week, calendar month, calendar year, and county fixed effects.

Statistical inference was based on cluster-robust standard errors at the meteorological-station level [25]. Because only ten meteorological-station clusters were available, additional station-level cluster bootstrap estimation with 999 resamples was performed to provide robust confidence intervals [25].

Pre-specified sensitivity analyses included (i) the sustained-heat exposure definition, (ii) distributed-lag models evaluating associations across lags 0–3 days, and (iii) county-stratified models to assess geographical heterogeneity. The distributed-lag results and the additional sensitivity analyses are reported in the Supplementary Materials (File S1: Supplementary analysis report).

**Figure 1:**
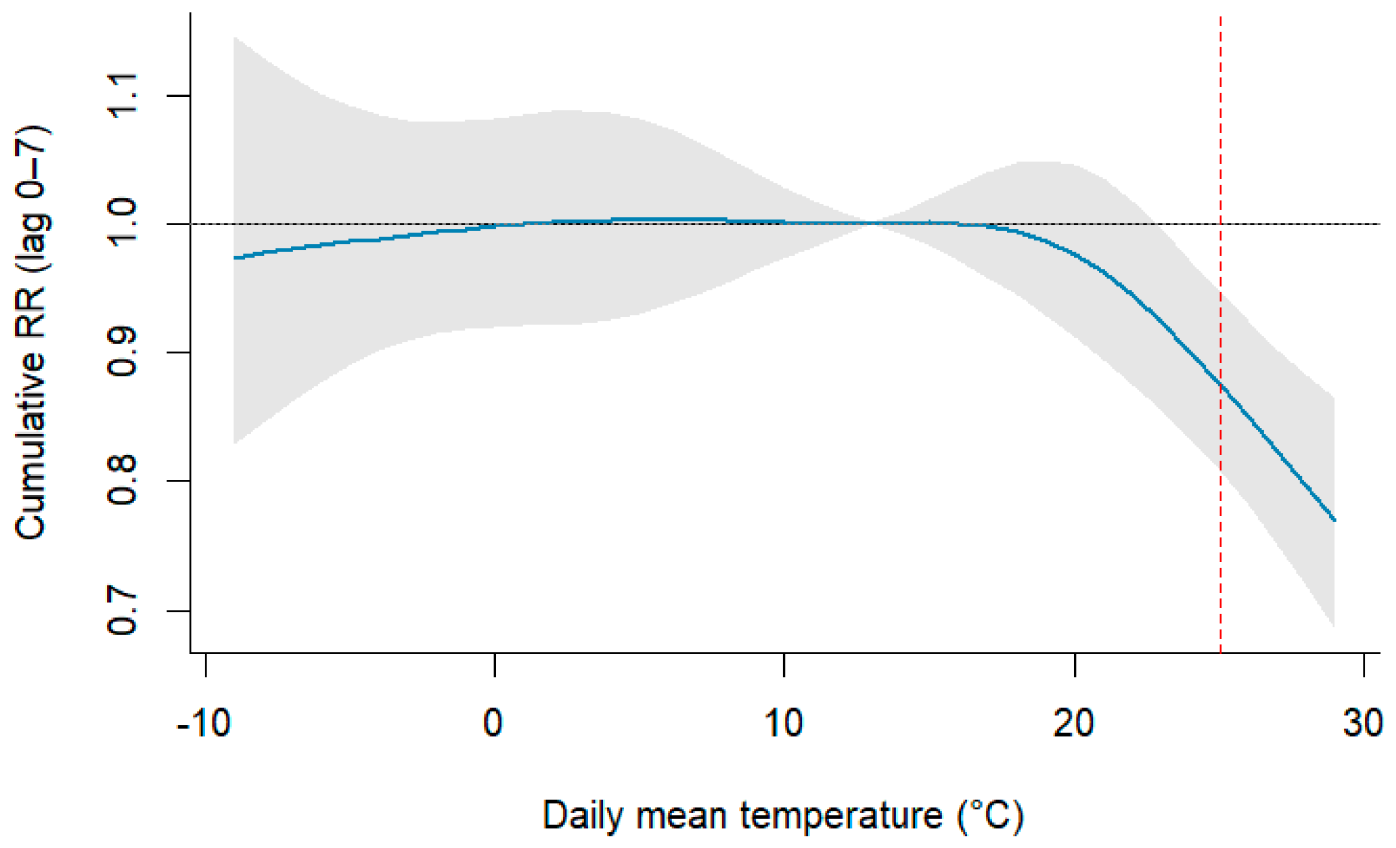
Continuous temperature exposure–response relationship for AMI admissions, derived from a distributed-lag non-linear model on the full county-day panel (lag 0–7 days; reported as exploratory, not the primary analysis). The reference temperature is the median daily mean temperature in the analytic panel, 13.1 °C. The shaded band shows the 95% pointwise confidence interval.

The binary heat-alert-threshold indicator was used as the primary exposure because it mirrors the real-world, policy-relevant exposure of direct public-health interest—a threshold that triggers alert issuance—while the continuous-temperature DLNM (Figure 1) served as a dose–response-sensitive complement rather than a substitute. The 0–3-day primary lag window was likewise chosen a priori to test same-day and short-delay physiological plausibility (acute triggering rather than delayed presentation); the longer 0–7-day exploratory DLNM was fitted specifically to assess whether associations extended beyond this window, and its lag structure—strongest at lag 0 with attenuation thereafter—is consistent with the primary model rather than contradicting it.

As a supplementary exploratory analysis, a distributed-lag non-linear model (DLNM) was fitted on the full county-day panel to characterize the continuous temperature–admission relationship across lags 0–7 days. Natural cubic splines were used for both the exposure–response and lag–response dimensions, with adjustment for the same covariates included in the primary model. The resulting cumulative exposure–response curve is presented in Figure 1. These analyses were exploratory and were not used as the primary estimate of the heat-alert-threshold effect. Because this analysis remains conditional on hospital admission, any apparent reduction in admission counts at the upper tail of the temperature distribution must be interpreted in light of the prehospital competing-risk mechanisms discussed in Section 4.2.

#### 2.6.3. Cumulative Mortality Analysis

All-cause mortality was analyzed at the patient level using Cox proportional hazards regression restricted to one index event per patient, with time from index admission as the time scale. Tied event times were handled using Efron’s method. The proportional hazards assumption was assessed using Schoenfeld residual tests. Several covariates violated the proportional hazards assumption in unstratified models, whereas the heat-alert-threshold exposure did not.

The primary adjusted model included age, sex, diabetes mellitus, heart failure, smoking status, prior coronary artery bypass grafting, and calendar year of index event. Infarction type, prior MI, hypertension, and prior PCI were incorporated as stratification variables to account for non-proportional hazards. An extended sensitivity model additionally adjusted for procedural variables available in the registry, including percutaneous coronary intervention, coronary angiography, and cardiac catheterization at the index event.

Pre-specified sensitivity analyses included (i) restriction to summer index events (June–August), (ii) substitution of the sustained-heat exposure definition, and (iii) time-stratified models estimating separate hazard ratios across follow-up intervals (0–30 days, 31–365 days, and >365 days). Pre-specified subgroup analyses examined effect modification according to age (<65 vs. ≥65 years), sex, infarction type, diabetes status, and prior MI. Subgroup-specific hazard ratios were estimated using the primary stratified Cox model fitted separately within each subgroup.

#### 2.6.4. Software and Reproducibility

All analyses were conducted in R version 4.5.2 [27] using standard survival, regression, and distributed-lag non-linear modeling packages. Cluster bootstrap confidence intervals were estimated using station-level resampling with replacement. The analysis pipeline is reproducible from the cleaned analytic dataset, and the corresponding R scripts are available from the corresponding author upon reasonable request.

## 3. Results

### 3.1. Cohort Characteristics

Between 1 January 2018 and 31 December 2019, the Hungarian Myocardial Infarction Registry recorded 30,883 AMI events from 29,596 unique patients. The mean age of the cohort was 67.2 (SD 12.8) years, 60.3% were male, and index events were classified as STEMI in 42.0% and NSTEMI in 58.0% of cases. The most prevalent comorbidities were hypertension (80.3%), diabetes mellitus (35.4%), and prior MI (23.5%), while 36.6% were current or former smokers (Table 1). Coronary angiography was performed in 85.7% of cases and PCI in 69.7%.

Of all admissions, 1287 (4.2%) represented recurrent AMI events. A total of 1933 events (6.3%) occurred on heat-alert-threshold days, concentrated predominantly between June and August. Baseline characteristics were well balanced between exposure groups (all SMDs < 0.10) (Table 1). The largest observed imbalance was for diabetes prevalence (SMD 0.051). Median follow-up after the index event was 763 days (maximum 1262 days). During follow-up, 8887 deaths (28.8%) occurred across the full cohort and 8420 deaths (28.4%) among the unique-patient index events included in the survival analysis.

### 3.2. Heat-Alert-Threshold Days and AMI Admissions

Within the summer-restricted county-day panel (3429 county-days, including 809 heat-alert-threshold county-days), AMI admissions were lower on heat-alert-threshold days than on non-alert summer days. The crude summer-only admission rate ratio was 1.155 (95% CI 0.993–1.343; *p* = 0.061). After adjustment for day-of-week, county, calendar year, and calendar month, the association reversed direction and remained consistently below 1.0 across adjusted models, with the fully adjusted model yielding an aIRR of 0.933 (95% CI 0.905–0.961; *p* < 0.001) (Table 2). Station-level cluster bootstrap analysis produced a similar estimate (aIRR 0.933; 95% CI 0.895–0.969), consistent with the primary cluster-robust analysis (Table 2).

**Figure 2:**
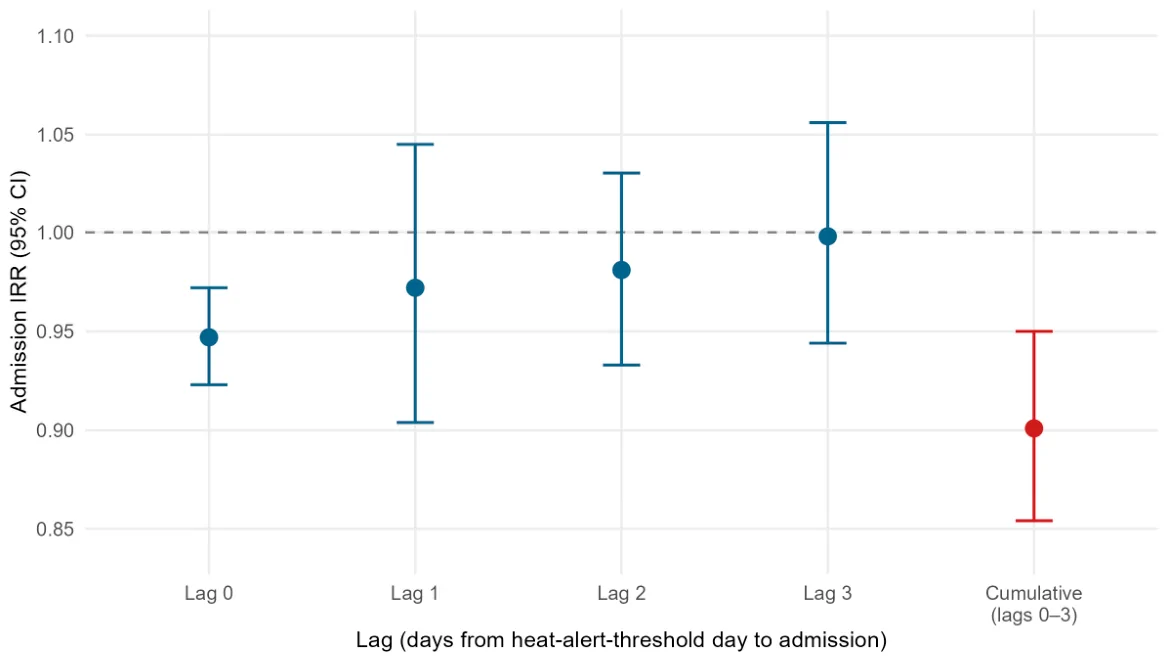
Distributed-lag aIRRs for heat-alert-threshold days on AMI admission counts at lags 0 through 3 days plus the cumulative effect across lags 0–3; summer-only county-day Poisson model. Vertical bars show 95% confidence intervals; the horizontal reference line is at IRR = 1.

**Figure 3:**
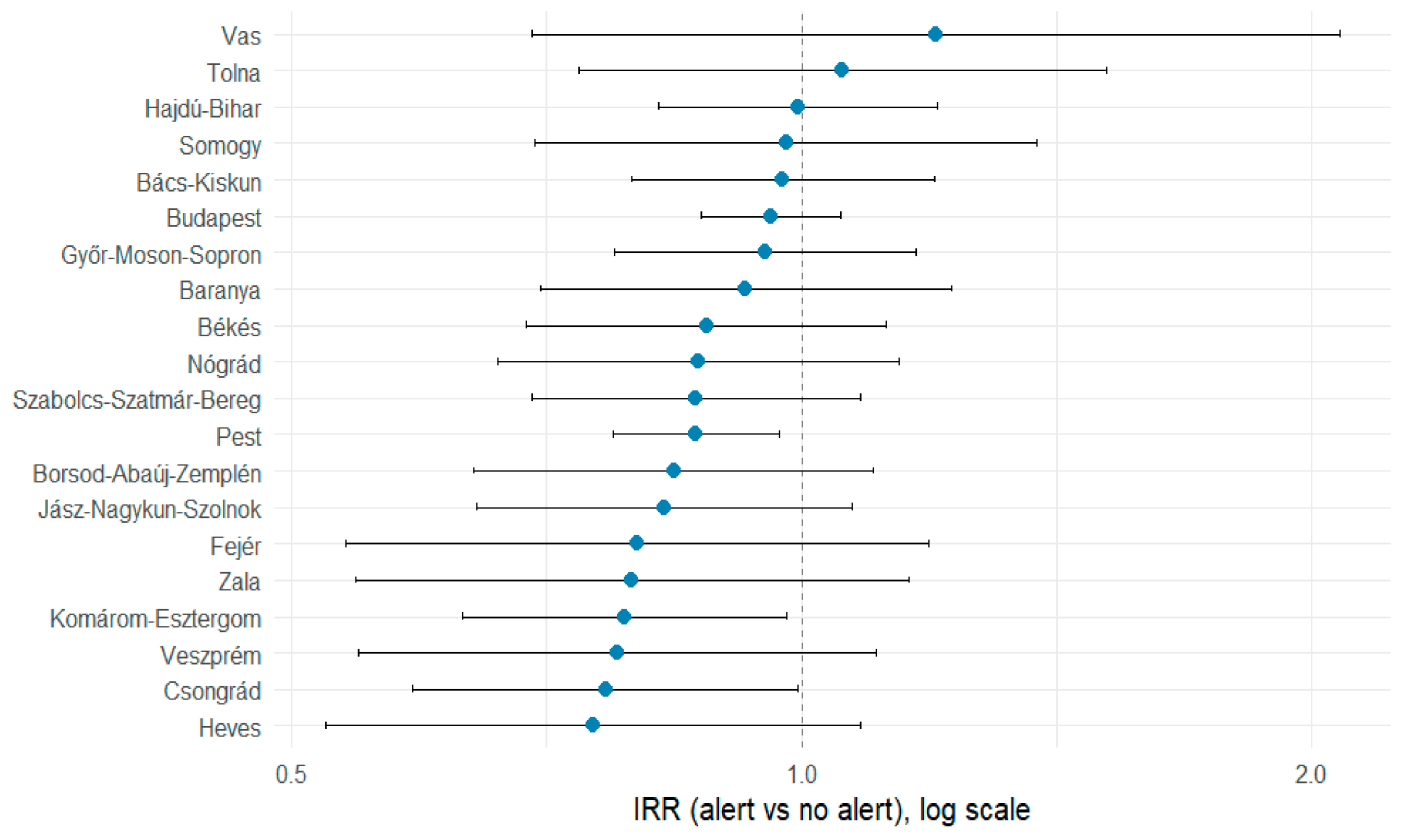
County-specific heat-alert-threshold aIRRs for AMI admissions, with 95% confidence intervals, ordered by point estimate. Each county-specific estimate is from an independent quasi-Poisson model adjusted for time trend and day-of-week within that county.

Sensitivity analyses yielded similar results. The sustained heat exposure definition was associated with an aIRR of 0.945 (95% CI 0.908–0.983; *p* = 0.005). Distributed-lag analyses demonstrated the strongest association at lag 0 (aIRR 0.947, 95% CI 0.923–0.972), whereas lag-1 to lag-3 estimates were not statistically significant (Figure 2). The cumulative IRR across lags 0–3 was 0.901 (95% CI 0.854–0.950) (Table 2). County-stratified analyses showed generally consistent estimates across counties, with most point estimates at or below 1.0 (Figure 3). Wider confidence intervals were observed in counties with smaller event counts.

### 3.3. Heat-Alert-Threshold Days and Cumulative Mortality After AMI

**Figure 4:**
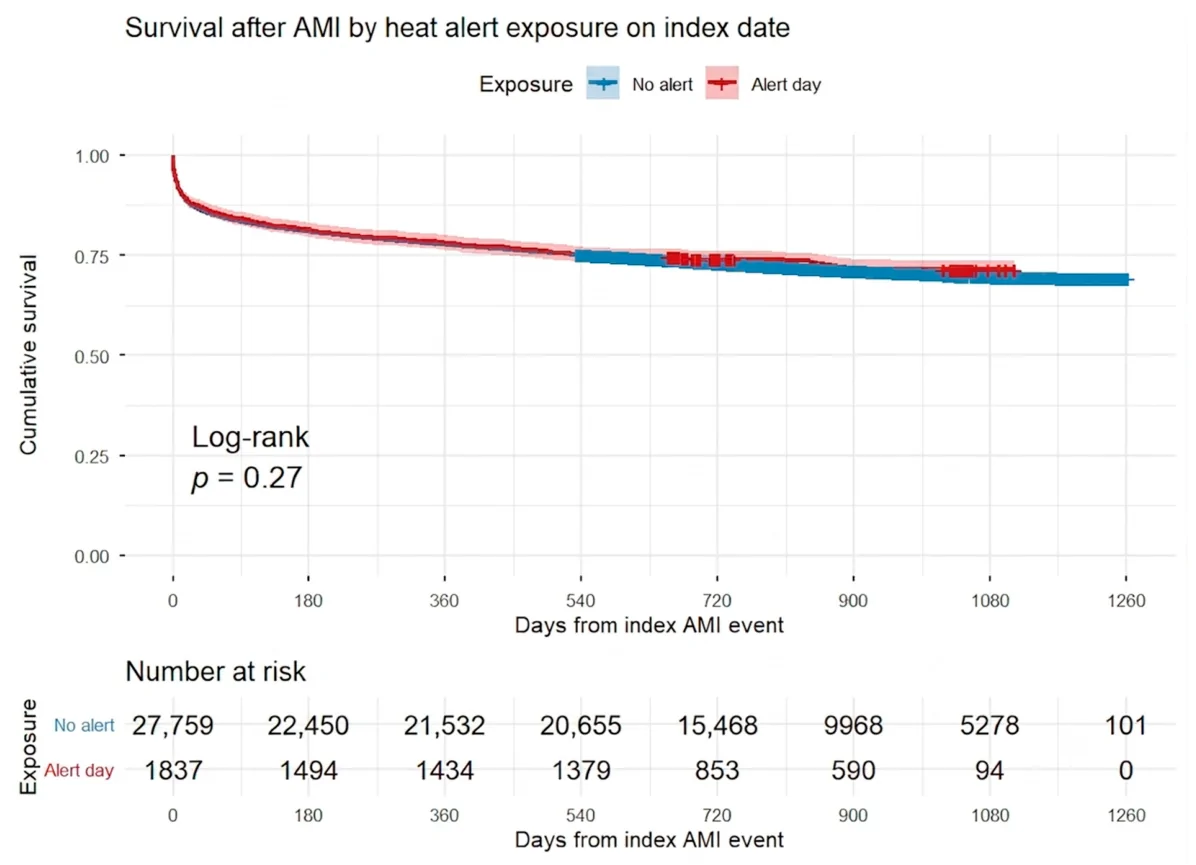
Kaplan–Meier survival curves for cumulative all-cause mortality after the index AMI event, stratified by heat-alert-threshold exposure on the day of admission. Solid lines show point estimates; shaded bands show 95% pointwise confidence intervals. The number-at-risk table at the foot of the figure shows the number of patients still under observation at each landmark. Log-rank test *p* = 0.27.

Among the 29,596 unique-patient index events, 8420 deaths (28.4%) occurred during follow-up. Kaplan–Meier survival curves showed no meaningful separation between patients admitted on heat-alert-threshold days and those admitted on non-alert days (log-rank *p* = 0.27; Figure 4). In the primary stratified Cox proportional hazards model, adjusted for demographic and clinical covariates and stratified by infarction type, prior MI, hypertension, and prior PCI, heat-alert-threshold exposure on the admission day was not associated with subsequent all-cause mortality (aHR 0.957, 95% CI 0.874–1.049; *p* = 0.35) (Table 3). The proportional hazards assumption was satisfied for the heat-alert-threshold exposure.

Conventional prognostic factors behaved as expected, with higher mortality associated with increasing age, heart failure, diabetes, and prior MI, whereas prior PCI and NSTEMI were associated with lower mortality. The model concordance index was 0.725. Results were consistent across sensitivity analyses. In the extended model additionally adjusting for procedural variables, the heat-alert hazard ratio was 0.937 (95% CI 0.855–1.026; *p* = 0.16). Restriction to summer-only index events yielded an aHR of 1.003 (95% CI 0.904–1.113), while substitution of the sustained-heat exposure definition produced similarly null estimates. Time-stratified analyses demonstrated no significant association between heat-alert-threshold exposure and mortality across the early (0–30 days), intermediate (31–365 days), or late (>365 days) follow-up periods (Table 3).

### 3.4. Subgroup Analyses

**Figure 5:**
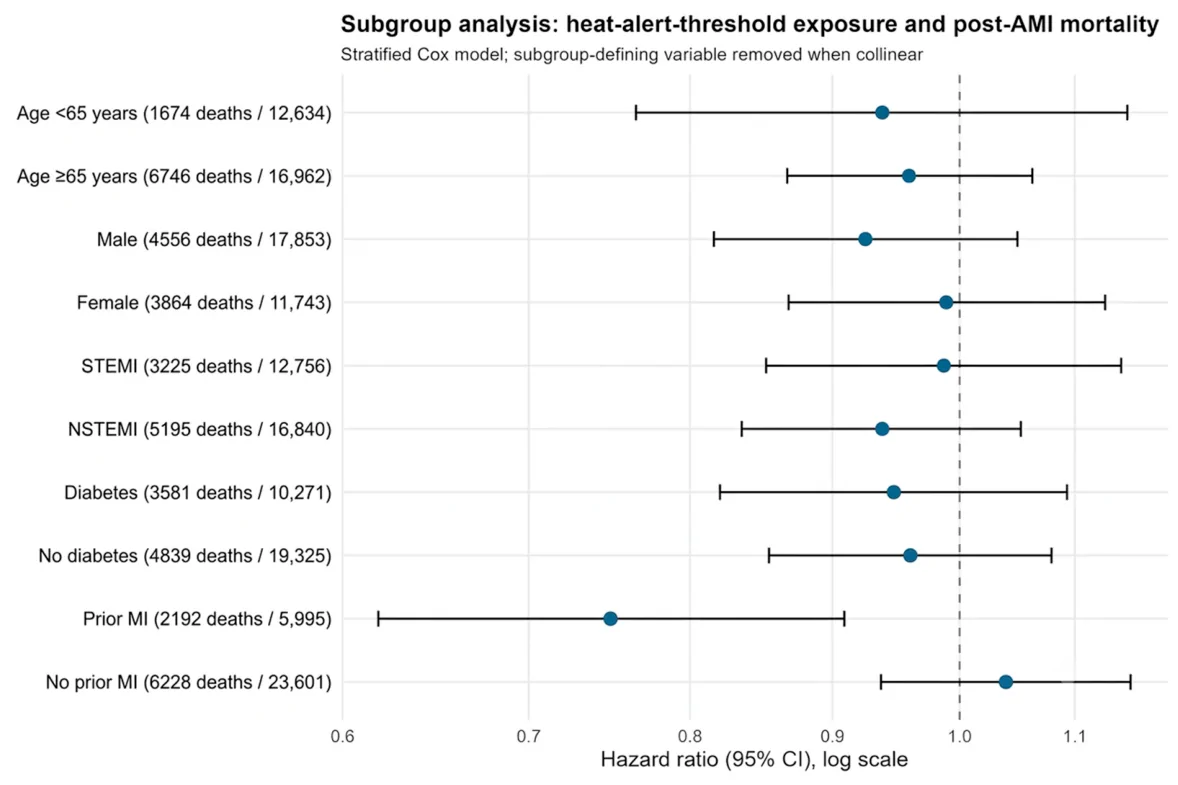
Forest plot of subgroup-specific adjusted hazard ratios for the heat-alert-threshold exposure (the blue dots), with 95% confidence intervals (the black lines). Each row shows a single subgroup; subgroup-specific Cox models drop the subgroup-defining variable from the covariate set when it would be collinear within the subgroup.

Pre-specified subgroup analyses showed broadly consistent null associations across age, sex, infarction type, and diabetes status (Table 4). The only subgroup with a non-null estimate was patients with prior MI (*n* = 5995; 2192 deaths), among whom heat-alert-threshold exposure was associated with lower mortality (aHR 0.749, 95% CI 0.618–0.909; *p* = 0.003). In patients without prior MI, the corresponding estimate was 1.039 (95% CI 0.937–1.152; *p* = 0.47) (Figure 5). Because multiple subgroup analyses were performed and the finding was not consistently observed across other clinical strata, this result should be interpreted as exploratory and hypothesis-generating. Five pre-specified subgroup analyses were performed without correction for multiple comparisons; the prior-MI subgroup finding should therefore not be used to guide clinical risk stratification without independent confirmation.

## 4. Discussion

In this nationwide registry-based study, heat-alert-threshold days were associated with fewer recorded AMI admissions during summer, whereas no association was observed between heat-alert-threshold exposure and subsequent all-cause mortality among hospitalized AMI patients. These findings remained consistent across multiple sensitivity analyses, including sustained-heat definitions, summer-restricted models, and time-stratified analyses. Patients admitted on heat-alert-threshold days had baseline clinical characteristics similar to those admitted on non-alert days at baseline.

### 4.1. Comparison with Published Literature

Our admissions findings differ from much of the existing heat–cardiovascular literature, which has generally reported increased cardiovascular morbidity and mortality during periods of extreme heat [11,13,14,28]. Accordingly, the present findings should be interpreted as hypothesis-generating rather than contradictory to the broader body of evidence. However, prior studies have varied substantially in exposure definitions, lag structures, climatic settings, and outcome ascertainment, and some studies have reported weaker or null associations between high temperature and AMI admissions [16]. Importantly, most previous investigations evaluated ambient temperature directly rather than temperature-defined heat-alert-threshold exposure within the context of national heat-health action plans.

For example, Bhaskaran et al.’s hourly-level case-crossover analysis of the UK MINAP registry and meta-analyses by Cheng et al. and Liu et al. reported increased cardiovascular morbidity with heat exposure using individual-level or fine-grained temporal exposure ascertainment, in contrast to the county-day ecological exposure and hospital-conditional outcome definition used here; we offer this as a methodological, rather than purely geographic, explanation for the divergence. Although older age and NSTEMI presentation have been reported elsewhere as heat-vulnerability markers, we did not observe effect modification by age (≥65 vs. <65 years) or infarction type (NSTEMI vs. STEMI) in the pre-specified subgroup analyses (Table 4). Given modest event counts within strata and the same competing-risk ascertainment mechanism proposed to operate across the whole cohort, these null subgroup results should not be read as evidence against differential heat vulnerability by age or infarction type, but as consistent with a hospital-admission-conditional design that may mask such differences irrespective of subgroup.

### 4.2. Pathophysiological Mechanisms Underlying the Inverse Admissions Signal

The observed inverse association between heat-alert-threshold days and recorded AMI admissions should not be interpreted as evidence of a protective effect of heat exposure. One epidemiologically and biologically plausible explanation is that heat stress may disproportionately precipitate fatal prehospital cardiovascular events that are not captured in a hospital admission registry, including out-of-hospital cardiac arrest, sudden cardiac death, and severe heat-related cardiovascular collapse [8,10,11,12,13]. Such events may reduce the number of patients surviving to hospital admission despite an unchanged or even increased underlying cardiovascular event burden. Alternative explanations include residual exposure misclassification, delayed care-seeking, unmeasured environmental confounding, and collider bias arising from conditioning the analysis on hospitalized patients. Because the present study was not designed to distinguish between these potential mechanisms, these explanations should be regarded as hypotheses requiring confirmation in future studies integrating hospital, emergency medical service, and out-of-hospital mortality data.

### 4.3. Mortality Displacement and the Hospitalization-Versus-Population Question

Critically, the present admission analysis was conditional on hospital presentation with an I21–I22 diagnosis. The HMR captures only AMI events for which the patient survived hospital admission and received a discrete AMI code. Out-of-hospital cardiac arrests, sudden cardiac deaths, ventricular arrhythmia deaths, heat-stroke-related cardiovascular fatalities, and silent infarctions are not included in the registry. Population-based studies of out-of-hospital cardiac arrest and cardiovascular mortality during heatwaves consistently report increases in fatal prehospital events [9,11,13]. Our results therefore should not be interpreted as evidence that the overall cardiovascular burden of heat in the Hungarian population is unchanged or reduced; the most pathophysiologically consistent interpretation of the inverse admissions signal is that heat-related cardiovascular catastrophe may occur outside the registry’s ascertainment window.

Short-term mortality displacement (“harvesting”) is a recognized phenomenon in heat-mortality epidemiology [29,30,31]. Because our mortality analysis evaluated long-term outcomes after hospitalization, short-term heat-related effects occurring over days or weeks may not necessarily be reflected in cumulative hazard ratios over prolonged follow-up. Accordingly, the null mortality association observed in this study may reflect either the absence of a measurable effect among hospitalized AMI patients or the averaging-out of short-term mortality displacement over time.

### 4.4. Implications

Our findings suggest that temperature-defined heat-alert-threshold days in Hungary were not associated with increased recorded AMI admissions or adverse long-term prognosis among hospitalized AMI patients. However, the observational design and the absence of direct linkage to administrative heat-alert records preclude causal inference regarding the effectiveness of heat-health action plans.

Because administrative alert declarations were not linked to the analytic dataset, this study cannot speak to the real-world public-health value of the alert system itself—only to the temperature threshold used to trigger it—and should not be cited as evidence for or against the effectiveness of Hungary’s heat-health action plan. The null mortality result applies only to patients who survived hospitalization and should not be read as reassurance that heat exposure carries no cardiovascular mortality risk at the population level; it instead underscores that risk stratification, and prevention efforts must extend beyond hospitalized cohorts. Integration of hospital registries with emergency-medical-services and out-of-hospital cardiac arrest data is a prerequisite, not merely a refinement, for accurately characterizing the cardiovascular burden of heat exposure.

## 5. Conclusions

In this nationwide Hungarian registry-based study, temperature-defined heat-alert-threshold days were associated with fewer recorded AMI admissions during summer, whereas no association was observed with subsequent long-term all-cause mortality among hospitalized AMI patients. The observed inverse association should not be interpreted as evidence of a protective effect of heat exposure. Alternative explanations include behavioral adaptation, delayed care-seeking, exposure misclassification, residual confounding, and the possibility of unmeasured out-of-hospital cardiovascular events that were not captured by the registry. Because the present study was not designed to distinguish between these potential mechanisms, these explanations should be regarded as hypotheses requiring further investigation. The null mortality finding applies only to patients who survived hospital admission and should not be interpreted as a population-level estimate of cardiovascular mortality during periods meeting the heat-alert temperature criterion. Future studies integrating the Hungarian Myocardial Infarction Registry with emergency medical services, out-of-hospital cardiac arrest registries, national mortality data, and administrative heat-alert records are needed to better understand the cardiovascular impact of extreme heat and to evaluate the effectiveness of heat-health action plans.

### 5.1. Study Strengths

Principal strengths of the study include the nationwide coverage and completeness of the Hungarian Myocardial Infarction Registry [23], deterministic linkage to the Central Death Registry, separate analytical frameworks for admission and mortality outcomes, and the use of station-level cluster bootstrap inference to address the small number of meteorological clusters [25].

### 5.2. Future Research Directions

Future studies should integrate hospital registry data with emergency-medical-services records and community mortality databases to better characterize out-of-hospital cardiovascular events during heat episodes. Direct linkage to administrative heat-alert records, including alert timing and intensity, would allow more rigorous evaluation of heat-alert implementation. In addition, individual-level exposure assessment using residential geocoding, indoor temperature monitoring, or wearable devices may help reduce exposure misclassification inherent to registry-based ecological analyses. Further, the air pollution parameters, in particular, PM_25_, ozone (O_3_), NO_2_, and other pollutants, should be included and linked to the dataset to see the effect with and without these parameters. In addition to that, sensitivity analyses across multiple temperature thresholds as a named follow-up should be done.

### 5.3. Limitations

Several limitations should be considered. First, the exposure variable was defined using station-level temperature data and the temperature threshold associated with Hungarian heat-alert issuance, but administrative heat-alert declarations were not directly linked to the analytic dataset. Accordingly, the exposure should be interpreted as a temperature-defined operational indicator rather than a direct measure of heat-health action plan implementation. Second, exposure assignment was based on the meteorological station linked to each patient’s residential county and therefore did not capture individual-level variation in environmental exposure, including urban heat-island effects, indoor climate, air-conditioning use, or personal activity patterns. Residual confounding by co-occurring environmental exposures, particularly air pollution, could not be excluded. Third, the registry did not contain several clinically important markers of AMI severity that may influence long-term prognosis. Fourth, the admission outcome captured only hospital-presenting AMI events and did not include out-of-hospital cardiac fatalities or silent infarctions. Fifth, the analytic period included only two summers, and replication using additional registry years would strengthen the assessment of temporal stability. Finally, this was a single-country observational study, and generalizability to other healthcare systems, climates, and populations should be interpreted cautiously. Because the analyses were restricted to hospitalized patients, collider bias cannot be excluded. Both heat exposure and disease severity may influence the probability of hospital admission, potentially affecting the observed associations among hospitalized individuals.

The ecological, station-level exposure assignment described above does not capture urban heat-island effects, altitude differences, building density, or green-space availability, all of which can produce meaningful within-county exposure heterogeneity. The residual confounding by air pollution noted above specifically concerns fine particulate matter (PM_2.5_), ozone (O_3_), and nitrogen dioxide (NO_2_), which covary with ambient temperature and independently affect cardiovascular risk; daily station-level concentrations of these pollutants were not available for linkage in the present analysis. Mean daily temperature also does not capture the contribution of humidity, wind speed, or solar radiation to physiological heat stress; composite indices such as the Heat Index, Wet-Bulb Globe Temperature, or Universal Thermal Climate Index were not available for this analysis and represent a priority for future work. Finally, because long-term (median 763-day) mortality follow-up may average short-term heat-related mortality displacement (“harvesting”) toward the null, the null adjusted hazard ratio reported here should not be interpreted as evidence against a short-term mortality effect of heat exposure.

## Figures and Tables

**Table 1:** Baseline characteristics of the cohort by heat-alert-threshold exposure on the index admission day.

Characteristic	Overall (*n* = 30,883)	No Alert (*n* = 28,950)	Alert Day (*n* = 1933)	SMD
Age, years (mean ± SD)	67.2 ± 12.8	67.2 ± 12.8	67.0 ± 13.4	0.018
Male sex, *n* (%)	18,631 (60.3)	17,452 (60.3)	1179 (61.0)	0.015
Infarction type, *n* (%)				
STEMI	12,973 (42.0)	12,168 (42.0)	805 (41.6)	0.008
NSTEMI	17,910 (58.0)	16,782 (58.0)	1128 (58.4)	
Hypertension, *n* (%)	24,786 (80.3)	23,251 (80.3)	1535 (79.4)	0.023
Diabetes mellitus, *n* (%)	10,919 (35.4)	10,279 (35.5)	640 (33.1)	0.051
Prior MI, *n* (%)	7247 (23.5)	6788 (23.4)	459 (23.7)	0.007
Heart failure, *n* (%)	4910 (15.9)	4606 (15.9)	304 (15.7)	0.005
Current/ex-smoker, *n* (%)	11,298 (36.6)	10,585 (36.6)	713 (36.9)	0.007
Prior PCI, *n* (%)	6490 (21.0)	6081 (21.0)	409 (21.2)	0.004
Prior CABG, *n* (%)	1767 (5.7)	1655 (5.7)	112 (5.8)	0.003

SMD = standardized mean difference; values > 0.10 indicate meaningful imbalance.

**Table 2:** AMI admission incidence rate ratios for heat-alert-threshold days versus non-alert days; summer-restricted county-day Poisson models.

Model	aIRR	95% CI	*p*-Value
Crude (no adjustment)	1.155	0.993–1.343	0.061
Adjusted: +day-of-week + county	0.920	0.895–0.947	<0.001
Adjusted: +year	0.923	0.898–0.947	<0.001
Primary: +month (full adjustment)	0.933	0.905–0.961	<0.001
Primary, station-level cluster bootstrap (999 reps)	0.933	0.895–0.969	<0.001
Sensitivity: sustained-heat exposure	0.945	0.908–0.983	0.005
Distributed lag, lag 0	0.947	0.923–0.972	<0.001
Distributed lag, lag 1	0.972	0.904–1.045	0.43
Distributed lag, lag 2	0.981	0.933–1.030	0.44
Distributed lag, lag 3	0.998	0.944–1.056	0.95
Distributed lag, cumulative (lags 0–3)	0.901	0.854–0.950	<0.001

aIRR = adjusted incidence rate ratio for AMI admissions. All adjusted models include day-of-week, county fixed effects, calendar year, and calendar month unless otherwise noted. Confidence intervals reflect cluster-robust standard errors at the meteorological-station level (*n* = 10) except where noted.

**Table 3:** Cox proportional hazards results for cumulative all-cause mortality, by exposure to heat-alert-threshold day on index admission.

Model	aHR	95% CI	*p*-Value
Primary stratified Cox (all index events)	0.957	0.874–1.049	0.35
Primary, summer-only	1.003	0.904–1.113	0.96
Sustained-heat exposure (all events)	1.013	0.910–1.126	0.82
Sustained-heat exposure, summer-only	1.073	0.955–1.205	0.23
Extended (+PCI, angiography, catheterization)	0.937	0.855–1.026	0.16
Extended, summer-only	0.977	0.881–1.085	0.67
Time-stratified: 0–30 days	0.964	0.843–1.102	0.59
Time-stratified: 31–365 days	1.010	0.867–1.176	0.90
Time-stratified: >365 days	0.858	0.695–1.059	0.15

aHR = adjusted hazard ratio. All models stratify the baseline hazard on infarction type, prior MI, hypertension, and prior PCI to satisfy the proportional hazards assumption, and adjust for age, sex, diabetes, heart failure, smoking, prior CABG, and calendar year of index event. The extended model additionally adjusts for PCI, angiography, and catheterization at the index event.

**Table 4:** Subgroup-specific hazard ratios for cumulative all-cause mortality by heat-alert-threshold exposure.

Subgroup	*N*	Deaths	aHR	95% CI	*p*-Value
Age < 65 years	12,634	1674	0.938	0.765–1.149	0.54
Age ≥ 65 years	16,962	6746	0.959	0.867–1.062	0.42
Male	17,853	4556	0.925	0.816–1.049	0.22
Female	11,743	3864	0.989	0.868–1.128	0.87
STEMI	12,756	3225	0.987	0.852–1.143	0.86
NSTEMI	16,840	5195	0.938	0.835–1.052	0.27
Diabetes	10,271	3581	0.947	0.820–1.093	0.46
No diabetes	19,325	4839	0.960	0.854–1.079	0.50
Prior MI	5995	2192	0.749	0.618–0.909	0.003
No prior MI	23,601	6228	1.039	0.937–1.152	0.47

aHR = adjusted hazard ratio from a subgroup-specific stratified Cox model. The variable defining each subgroup was removed from the covariate set when collinear within that subgroup; otherwise, the covariate set is identical to the primary model.

## Data Availability

The cleaned analytic dataset is available in the Mendeley Data repository (DOI: 10.17632/2V7N2R3XCH.1).

## Supplementary Materials

The following supporting information can be downloaded at: https://www.mdpi.com/article/10.3390/ijerph23081010/s1. File S1: Supplementary analysis report.

## Abbreviations

The following abbreviations are used in this manuscript:

AMI	Acute Myocardial Infarction
STEMI	ST-Elevation Myocardial Infarction
NSTEMI	Non-ST-Elevation Myocardial Infarction
NNK	National Public Health Center
HMR	Hungarian Myocardial Infarction Registry
aIRR	Adjusted Incidence Rate Ratio
aHR	Adjusted Hazard Ratio
