# Supplementary material for: Temperature-Defined Heat-Alert-Threshold Days and Acute Myocardial Infarction Admissions and Mortality: A Nationwide Hungarian Registry-Based Cohort Study
Source: Int J Environ Res Public Health. 2026 Aug 2;23(8):1010. doi: 10.3390/ijerph23081010 (PMC13512502; doi:10.3390/ijerph23081010)
Supplement: Supplementary file 1 [file ijerph-23-01010-s001.zip › File S1 Supplementary analysis report.pdf]

Supplementary analysis report

S1. Threshold sensitivity (25–32°C, lag 0)

Across the full threshold ladder, the inverse association between heat-stress-day exposure and same-day AMI admissions was directionally consistent at every threshold tested, for both daily mean and daily maximum temperature. On daily maximum temperature — the metric with adequate exposure frequency across the whole 25–32°C range — the aIRR moved from 0.95 (95% CI 0.86–1.06) at 25°C to 0.89 (95% CI 0.86–0.93) at 32°C, with the association reaching statistical significance from 29°C upward. On daily mean temperature, which in the Hungarian summer climate rarely exceeds 29°C, the same-day association strengthened further at higher thresholds (0.89 at 25°C to 0.80 at 28°C); the 29°C cell (aIRR 0.61) is based on only 20 exposed county-days and is reported for completeness rather than as a stable estimate.

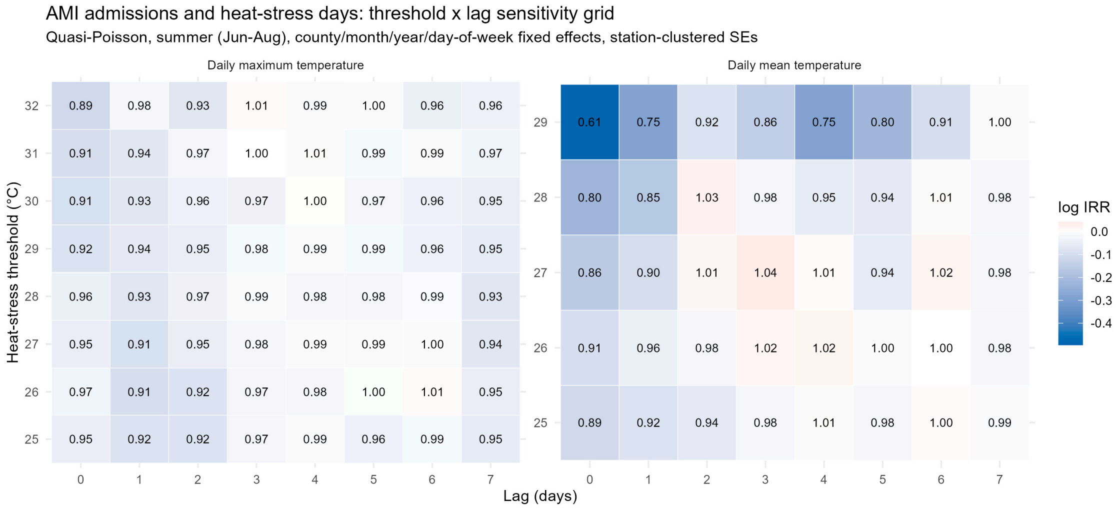

Figure S1. Full threshold × lag sensitivity grid (aIRR by cell). Colour reflects log(aIRR); cells below 1 (blue) indicate fewer admissions on heat-stress days. The daily-mean panel is restricted to 25–29°C because Hungarian summer daily means rarely reach 30°C. The two most saturated cells (mean ≥28°C and ≥29°C, lag 0) rest on only 49 and 20 exposed county-days respectively and should be read as illustrative of the gradient's direction rather than as precise point estimates.

Table S1. reports the corresponding point estimates and intervals for a representative subset of thresholds.

**Exposure (lag 0)**	**Exposed county-days**	**aIRR**	**95% CI**
Daily mean ≥25°C (primary, published)	871	0.89	0.85–0.94
Daily mean ≥26°C	437	0.91	0.84–0.99
Daily mean ≥27°C	177	0.86	0.82–0.90
Daily mean ≥28°C	49	0.80	0.78–0.82
Daily mean ≥29°C (n small — exploratory)	20	0.61	0.60–0.62
Daily max ≥25°C	3,211	0.95	0.86–1.06
Daily max ≥28°C	2,351	0.96	0.91–1.01
Daily max ≥30°C	1,582	0.91	0.87–0.96
Daily max ≥32°C	792	0.89	0.86–0.93

A false-discovery-rate correction (Benjamini–Hochberg) was applied across the full 104-cell threshold × lag grid; 32 of 104 cells remained significant after correction, and the pattern of attenuation with lag described below was unchanged.

S2. Lag-structured exposure (lag 1–7 days, evaluated separately)

For the primary exposure definition (daily mean ≥25°C), the inverse association was strongest at lag 0 and lag 2, attenuated toward the null by lag 3–4, and did not re-emerge at longer lags. The cumulative any-exposure indicator across lags 0–7 (i.e., at least one heat-stress day in the preceding week) reproduced an aIRR of 0.90 (95% CI 0.88–0.94), essentially unchanged from the single-day estimate. The same pattern — strongest effect at lag 0, attenuation thereafter — held for the higher daily-maximum thresholds (30–32°C), with one exception worth noting explicitly: at daily max ≥32°C, lag 1 showed a small *isolated positive association* above unity (aIRR 1.08, 95% CI 1.02–1.14) when all eight lags were entered simultaneously in a single distributed-lag model. This single reversal at one lag/threshold combination did not recur at neighbouring thresholds or lags and is discussed as an isolated finding rather than a competing signal.

Table S2. Lag-structured exposure

**Lag (days)**	**Mean ≥25°C, aIRR (95% CI)**	**Max ≥30°C, aIRR (95% CI)**	**Max ≥32°C, aIRR (95% CI)**
0	0.89 (0.84–0.97)	0.91 (0.89–0.97)	0.89 (0.85–0.96)
1	0.92 (0.88–1.00)	0.93 (0.90–1.02)	0.98 (0.95–1.08)
2	0.94 (0.91–0.99)	0.96 (0.91–1.00)	0.93 (0.85–0.98)
3	0.98 (0.89–1.06)	0.97 (0.91–1.01)	1.01 (0.93–1.11)
4	1.01 (0.94–1.08)	—	—
5	0.98 (0.93–1.02)	—	—
6	1.00 (0.94–1.07)	—	—
7	0.99 (0.92–1.02)	—	—
Cumulative, any exposure lags 0–7	0.90 (0.88–0.94)	0.96 (0.92–1.02)	0.92 (0.88–1.00)

95% CIs above are station-cluster bootstrap intervals (999 resamples) for the headline cells (lags 0–3 for max thresholds; lags 0–7 for the primary mean threshold), consistent with the inferential approach used for the primary published estimate.

S3. Consecutive (heat-wave-like) versus isolated heat-stress days

Distinguishing isolated single-day exposure from runs of two or more consecutive heat-stress days showed that the association is driven predominantly by sustained exposure rather than by isolated hot days. For the primary threshold (daily mean ≥25°C), isolated single-day exposure was not significantly associated with admissions (aIRR 0.91, 95% CI 0.75–1.04), whereas three or more consecutive heat-stress days showed a stronger and statistically significant inverse association (aIRR 0.87, 95% CI 0.78–0.94). A day-within-episode analysis (day 1, day 2, day 3+ of a run) showed the association was present from the first day of an episode and persisted through its duration, rather than emerging only later in a run. For daily max ≥32°C the pattern was reversed — isolated days showed the stronger association (aIRR 0.75, 95% CI 0.62–0.83) while 3+ consecutive days were

not significant (aIRR 0.94, 95% CI 0.89–1.09) — which may reflect the considerably smaller number of exposed county-days rather than a fundamentally different biological mechanism. All exposure categories retained at least 49 county-days, above the threshold we set a priori for interpretability.

Table S3. Consecutive (heat-wave-like) versus isolated heat-stress days

**Contrast (vs. no heat-stress day)**	**Exposed county-days**	**aIRR**	**95% CI**
Isolated day, mean ≥25°C	106	0.91	0.75–1.04
3+ consecutive days, mean ≥25°C	637	0.87	0.78–0.94
Isolated day, max ≥32°C	144	0.75	0.62–0.83
3+ consecutive days, max ≥32°C	489	0.94	0.89–1.09

S4. Summary and impact on conclusions

None of the three requested sensitivity analyses altered the direction, approximate magnitude, or interpretation of the primary finding. Across 25–32°C, at lags 1–7, and across isolated versus consecutive exposure, the estimated association between heat-stress-day exposure and recorded AMI admissions remained inverse and, at the thresholds and lags with adequate exposure frequency, statistically significant — consistent with the manuscript's original interpretation that this reflects under-ascertainment of prehospital events and related mechanisms discussed in Section 4.2, rather than a true protective effect of heat.

Overall, these additional analyses reinforce the robustness of the primary findings. The association is concentrated at lag 0–2 and attenuates at longer lags, arguing against a delayed-presentation explanation, and it is markedly stronger for sustained (3+ day) heat episodes than for isolated hot days at the primary threshold, consistent with a cumulative physiological or ascertainment mechanism rather than an artifact of a single arbitrarily chosen threshold. We are glad to incorporate the full threshold × lag grid (Supplementary Table), the lag-specific forest plot, and the consecutive-versus-isolated comparison as supplementary material in the revised manuscript, and to add a short paragraph to Section 4 summarizing these robustness checks.
