# Supplementary material for: Temperature-Defined Heat-Alert-Threshold Days and Acute Myocardial Infarction Admissions and Mortality: A Nationwide Hungarian Registry-Based Cohort Study
Source: Int J Environ Res Public Health. 2026 Aug 2;23(8):1010. doi: 10.3390/ijerph23081010 (PMC13512502; doi:10.3390/ijerph23081010)
Supplement: Supplementary file 1 [file ijerph-23-01010-s001.zip › Reporting Checklist.pdf]

The Reporting Checklist

Item	RECORD/STROBE element	Page / section in manuscript
**Title and abstract**		
1 (STROBE)	Indicate study design with a commonly used term in title or abstract	Title ("Cohort Study"); Abstract Methods ("nationwide registry-based study": county-day time-series of admissions and patient-level survival cohort)
1.1 (RECORD)	The type of data used should be specified in the title or abstract; the name of the database should be specified	Title and Abstract: "A Nationwide Hungarian Registry-Based Cohort Study"; Abstract: "Hungarian Myocardial Infarction Registry (HMR)"
1.2 (RECORD)	If applicable, the geographic region and timeframe within which the study took place should be reported in the title or abstract	Title: "Hungarian"; Abstract: "in Hungary"; Abstract: study period 1 January 2018–31 December 2019, mortality follow-up to 16 June 2021
1.3 (RECORD)	If linkage between databases was conducted for the study, this should be clearly stated in the title or abstract	Reported in Methods 2.5: HMR were deterministically linked to the Central Death Registry for vital-status follow-up; Methods 2.4: each patient county linked to nearest OMSZ meteorological station for exposure assignment
**Introduction**		
2 (STROBE)	Background, rationale	1 (Introduction), paragraphs 1–4
3 (STROBE)	Objectives	1 (Introduction), paragraph 5 (final paragraph): aims to examine AMI admission rates and cumulative all-cause post-AMI mortality, plus exploratory subgroup differences
**Methods**		
4 (STROBE)	Study design	2.1: nationwide retrospective registry-based study combining a county-day time-series analysis of AMI admissions with a patient-level cohort analysis of cumulative all-cause mortality; RECORD/STROBE reporting
5 (STROBE)	Setting	2.1, 2.2, 2.4 (19 counties plus Budapest; 10 OMSZ stations)
6 (STROBE)	Participants — eligibility criteria	2.3 (all HMR AMI events, 1 January 2018–31 December 2019; index event = first observed AMI per patient)
6.1	The methods of study	2.3: registry inclusion criteria — age ≥ 18

Item	RECORD/STROBE element	Page / section in manuscript
(RECORD)	population selection (e.g., codes or algorithms used to identify subjects) should be listed in detail	years, documented AMI (ICD-10 I21–I22), complete demographic data; index event defined as first observed AMI per patient during the study period
6.2 (RECORD)	Any validation studies of the codes or algorithms used to select the population should be referenced	Methods 2.2: HMR described in detail previously (Jánosi, 2024)
6.3 (RECORD)	If validation was conducted for this study, it should be described and the validation results should be reported	Not applicable — no de novo validation performed; existing HMR validation cited
7 (STROBE)	Variables — outcomes, exposures, predictors, potential confounders, effect modifiers	2.4 (exposure: heat-alert-threshold day; sustained-heat sensitivity definition), 2.5 (co-primary outcomes), 2.6.1 (cohort description), 2.6.2 and 2.6.3 (covariates, stratification variables, and effect modifiers)
7.1 (RECORD)	A complete list of codes and algorithms used to classify exposures, outcomes, confounders, and effect modifiers should be provided	Exposure: daily mean temperature ≥25 °C at assigned OMSZ station (2.4); outcomes: ICD-10 I21–I22 admission, deterministic linkage to Central Death Registry (2.5); exposure sensitivity definition: same-day and three-day rolling mean temperature > 25 °C (2.4); admission-model covariates: day-of-week, calendar month, calendar year, county fixed effects (2.6.2); Cox covariates: age, sex, diabetes mellitus, heart failure, smoking status, prior CABG, calendar year, with baseline hazard stratified on infarction type, prior MI, hypertension, and prior PCI (2.6.3); effect modifiers: age (< 65 vs. ≥ 65), sex, infarction type, diabetes, prior MI (2.6.3)
8 (STROBE)	Data sources / measurement	2.2 (HMR registry); 2.4 (OMSZ station temperature data); 2.5 (outcome ascertainment via Central Death Registry linkage)
9 (STROBE)	Bias	2.4 (ecological exposure assignment); 2.5 (admission outcome excludes out-of-hospital fatalities); 5.3 (Limitations — exposure misclassification, residual air-pollution confounding, collider bias from conditioning on hospitalization)
10 (STROBE)	Study size	2.3 (full cohort), 2.6.2 (county-day panel: 14,600 county-days), 3.1, 3.2 (3429 summer

Item	RECORD/STROBE element	Page / section in manuscript
		county-days, 809 alert days)
11 (STROBE)	Quantitative variables	2.4 (mean temperature dichotomized at ≥ 25 °C; sustained-heat definition), 2.6.1
12 (STROBE)	Statistical methods	2.6.1 (descriptive statistics, SMDs), 2.6.2 (quasi-Poisson county-day models; cluster-robust SEs and station-level cluster bootstrap, 10 clusters; distributed-lag and DLNM analyses), 2.6.3 (stratified Cox proportional hazards; sensitivity, time-stratified, and subgroup models), 2.6.4 (R version 4.5.2; reproducibility)
12.1 (RECORD)	Authors should describe the extent to which the investigators had access to the database population used to create the study population	Methods 2.2: dataset is publicly available on Mendeley (doi:10.17632/2V7N2R3XCH.1); authors had full access to the analytic dataset; Data Availability Statement
12.2 (RECORD)	Authors should provide information on the data cleaning methods used in the study	Methods 2.2 (standardized electronic case-report data capture); Methods 2.3 (minor inconsistencies identified during data cleaning were resolved prior to analysis without excluding any events)
12.3 (RECORD)	State whether the study included person-level, institutional-level, or other data linkage across two or more databases; the methods of linkage and methods of linkage quality evaluation should be provided	Methods 2.4: county-to-station geographic linkage of HMR events to OMSZ daily meteorological station data via county-to-station assignment; Methods 2.2 and 2.5: deterministic linkage of HMR to the National Health Insurance Fund database and to the Central Death Registry via unique patient identifiers; no formal linkage-quality evaluation (e.g., match rates) is reported
**Results**		
13 (STROBE)	Participants	3.1 (30,883 AMI events from 29,596 unique-patient index events; 1287 recurrent events, 4.2%); Table 1
13.1 (RECORD)	Describe in detail the selection of the persons included in the study (i.e., study population selection) including filtering based on data quality, data availability, and linkage. The selection of included persons can be described in the text and/or by means of the study	Methods 2.3 (eligibility criteria and data cleaning); Results 3.1 (cohort numbers: 30,883 AMI events from 29,596 unique patients)

Item	RECORD/STROBE element	Page / section in manuscript
	flow diagram	
14 (STROBE)	Descriptive data	Table 1; 3.1 (mean age 67.2 [SD 12.8] years; 60.3% male; STEMI 42.0% / NSTEMI 58.0%; all SMDs < 0.10, largest for diabetes 0.051; median follow-up 763 days, maximum 1262 days)
15 (STROBE)	Outcome data	3.2 (admissions: 1933 events, 6.3%, on heat-alert-threshold days); 3.3 (mortality: 8420 deaths, 28.4%, among unique-patient index events; 8887 deaths, 28.8%, in the full cohort)
16 (STROBE)	Main results	3.2 (summer admissions aIRR 0.93, 95% CI 0.90–0.97; sustained-heat aIRR 0.945, 95% CI 0.908–0.983), 3.3 (cumulative mortality aHR 0.96, 95% CI 0.87–1.05), 3.4 (subgroups); Tables 2–4; Figures 1–5
17 (STROBE)	Other analyses	Methods 2.6.2–2.6.3 (prespecified sensitivity and subgroup analysis plan); Results 3.2 (admission sensitivity and distributed-lag analyses), 3.3 (mortality sensitivity analyses), 3.4 (subgroup analyses); Supplementary Materials, File S1 (distributed-lag and additional sensitivity analyses)
**Discussion**		
18 (STROBE)	Key results	4 (opening paragraph); 5 (Conclusions)
19 (STROBE)	Limitations	5.3 (Limitations)
19.1 (RECORD)	Discuss the implications of using data that were not created or collected to answer the specific research question (s). Include discussion of misclassification bias, unmeasured confounding, missing data, and changing eligibility over time, as they pertain to the study being reported	5.3 (Limitations), specifically: exposure misclassification (station-level assignment), unmeasured confounders (severity markers, air pollution), and analysis confined to admitted events; missing data addressed by the registry's complete-demographic-data requirement, with no events excluded during cleaning (2.3), and eligibility criteria unchanged across the two-year study period. See also Section 4.2 (under-ascertainment / diagnostic reclassification of heat-related fatal events (cardiac arrest) rather than I21–I22) and 4.3 (hospitalization-vs-population scope)
20 (STROBE)	Interpretation	4.1 – 4.4; 5 (Conclusions)

Item	RECORD/STROBE element	Page / section in manuscript
21 (STROBE)	Generalisability	5.3 (Limitations) — single-country observational study, two summers only; 5.2 (Future Research Directions)
**Other information**		
22 (STROBE)	Funding	Funding section: “This research received no external funding”
22.1 (RECORD)	Authors should provide information on how to access any supplemental information such as the study protocol, raw data, or programming code	Methods 2.2 (analytic dataset publicly available in the Mendeley Data repository, DOI: 10.17632/2V7N2R3XCH.1); Methods 2.6.4 (R analysis scripts available from the corresponding author on reasonable request); Supplementary Materials statement (File S1: Supplementary analysis report); Data Availability Statement; RECORD checklist provided as Supplementary Material (stated in Methods 2.1)
